# Pathway Analysis Revealed Potential Diverse Health Impacts of Flavonoids that Bind Estrogen Receptors

**DOI:** 10.3390/ijerph13040373

**Published:** 2016-03-26

**Authors:** Hao Ye, Hui Wen Ng, Sugunadevi Sakkiah, Weigong Ge, Roger Perkins, Weida Tong, Huixiao Hong

**Affiliations:** Division of Bioinformatics and Biostatistics, National Center for Toxicological Research, U.S. Food and Drug Administration, AR 72079, USA; haoye.ecust@gmail.com (H.Y.); Huiwen.Ng@fda.hhs.gov (H.W.N.); Suguna.Sakkiah@fda.hhs.gov (S.S.); weigong.ge@fda.hhs.gov (W.G.); Roger.Perkins@fda.hhs.gov (R.P.); weida.tong@fda.hhs.gov (W.T.)

**Keywords:** estrogen receptor, flavonoids, target, gene, pathway analysis

## Abstract

Flavonoids are frequently used as dietary supplements in the absence of research evidence regarding health benefits or toxicity. Furthermore, ingested doses could far exceed those received from diet in the course of normal living. Some flavonoids exhibit binding to estrogen receptors (ERs) with consequential vigilance by regulatory authorities at the U.S. EPA and FDA. Regulatory authorities must consider both beneficial claims and potential adverse effects, warranting the increases in research that has spanned almost two decades. Here, we report pathway enrichment of 14 targets from the Comparative Toxicogenomics Database (CTD) and the Herbal Ingredients’ Targets (HIT) database for 22 flavonoids that bind ERs. The selected flavonoids are confirmed ER binders from our earlier studies, and were here found in mainly involved in three types of biological processes, ER regulation, estrogen metabolism and synthesis, and apoptosis. Besides cancers, we conjecture that the flavonoids may affect several diseases via apoptosis pathways. Diseases such as amyotrophic lateral sclerosis, viral myocarditis and non-alcoholic fatty liver disease could be implicated. More generally, apoptosis processes may be importantly evolved biological functions of flavonoids that bind ERs and high dose ingestion of those flavonoids could adversely disrupt the cellular apoptosis process.

## 1. Introduction

Flavonoids are a group of natural compounds sharing the basic parent structure of 2-phenyl-1,4-benzopyrone. They are widely distributed in modern human diets, including fruit, vegetable, tea and even wine [1,2], as they have been across geologic eras in the history of animals. A huge amount of literature supports the importance of flavonoids in the appearance and rise of higher primates. However, in the interest of human health, science must ask when there is too much of a good thing.

Flavonoids can be generally grouped into six chemical classes: flavones, flavonols, flavanones, flavanols, anthocyanidins and isoflavones [3]. Epidemiologic studies are strongly supportive that flavonoids-enriched diets promote health in diverse ways through diverse mechanisms—specifically, reduced cancer risk [4,5], antioxidation effects [6,7], mitigation of cardiovascular diseases [8,9], anti-inflammatory properties [10], counteraction of obesity [11], reinforce cognition [12], and potential addition to many beneficial effects of estrogen activity across tissue types [13,14]. Neither U.S. nor Eurozone regulators promulgate recommendations with respect to flavonoids, either official or unofficial. Regulatory decisions would require the same solid science basis and data as for drug clinical trials. Such study designs would normally be protracted and expensive and require rigor such as double-blind controlled experiments.

The supplement industry is a conglomeration of small to large companies comprising product pipelines. The companies are required to ensure that products are safe, but not to prove so with rigorous testing—so they do not. The Food and Drug Administration (FDA) will only take action if strong evidence appears of harm, or if specific marketing claims are deemed by the FDA to be untruthful and not misleading—see Dietary Supplement Health and Education Act of 1994 [15]. Thus, most claims are vague and generally in order to conform, while a vast amount of literature constituting conjecture, pseudoscience and even fraud is readily available to the public in venues from print to online that fosters self-medication, despite the Federal Trade Commission’s duty to control its truthfulness. Unfortunately, companies’ intent on deception can often deceive for a long and prosperous period of time. Regulatory authorities expend great effort to provide cautionary science-based education, but many consumers may simply hear what they want to hear in slick and obfuscatory off-label marketing. Recently, with academic studies raising alarms, some U.S. State Attorney Generals have initiated their own studies, with alarming findings that a large proportion of supplement products do not even contain the purported bioactive components, evidence that outright fraud is rampant. *Caveat emptor* seems to be the consumer’s smartest tactic when choosing supplements.

The health conscious public has become increasing aware of the potential health benefits of endocrine active chemicals such as the flavonoids. The active chemicals have been conserved across many plants and especially vertebrate metazoans through co-evolution. In certain cases, plants use isoflavones as biochemical defense weapons against predation. Not surprisingly, the market responded with various flavonoids compound formulations. In some formulations, ingested dose could be considered as high. For example, the recommended daily dose of quercetin supplement is up to 1136–2272 mg [16], compared to nominal total isoflavone in humans in 27.80 mg per day [17], the later common in high soy diets. Such a step increase in a bioactive chemical is justifiably concerning. Moreover, studies [18,19,20] have increased adverse effect concerns due to flavonoid-drug interactions. As a specific example, flavonoids may influence the multidrug resistance process through ATP-binding cassette (ABC) transporters [21]. Since the dietary supplements of flavonoids are not classified as drugs, their potential toxicities via interactions have not been adequately evaluated and are certainly needed.

The endocrine system contains glands that produce and release hormones that are carried to distant target organs in the body through circulation. A wide range of tissue-dependent biological responses are resulted from hormone binding endocrine receptor complexes, such as growth and development [22,23], vascular smooth muscle contraction [24] and reproduction [25]. Simply put, the normal endocrine system is paramount to coordinating and maintaining reproduction, development, wide-ranging body functions, and maintaining homeostasis in general. The so-called endocrine disruptors (EDs) are chemicals (normally exogenous) that interfere with normal endocrine function to an extent to cause adverse effects. Some scientists prefer using the terminology endocrine-active, but that may be drawing a distinction where there is no difference, as an exogenous hormone exposure superimposes on already active homeostatic hormone levels. Regardless whether you label them disruptors or active, significant concerns, public and regulatory, about EDs launched a major effort by the U.S. Environmental Protection Agency (EPA) named the Endocrine Disruptor Screening Program (EDSP) [26] to evaluate tens of thousands of natural and man-made chemicals for endocrine activity; many suspected industrial chemicals are lipophilic and produced in enormous quantity, ultimately persisting in food-chains worldwide. The estrogen receptors (ERs) are arguably the most important receptors in the endocrine system and are involved prodigious biological processes, and especially sensitive windows of time associated with reproduction and development to the adulthood system [27,28]. Many studies have shown that ERs are important to many mechanisms—transcription mediated or not—that exert therapeutic effects on neurodegenerative diseases [29,30], cancers [31,32], cardiovascular diseases [33,34], obesity [35,36] and osteoporosis [37], among others. It is hardly surprising then that homology and mutation rate studies infer that the early protein ancestors date to the early time on earth when metazoans were first arising. They are highly preserved across vertebrates. The awareness of the criticality of the nuclear receptors proteins prompted the U.S. FDA to build a comprehensive Estrogenic Activity Database (EADB) [38] in the Endocrine Disruptors Knowledge Base (EDKB) [39,40,41]. The aggregation of diverse assay data types into a single chemical-centric database was deemed essential to foster basic and regulatory science and the building of *in silico* predictive models for assessing endocrine activity in the FDA-regulated products and environmental chemicals [42,43,44,45,46,47,48]. Here, we utilize EADB curated data along with target genes’ data from public databases to more closely examine putative and possible beneficial and toxicity profiles of flavonoids that are ER binders.

## 2. Materials and Methods

### 2.1. Study Design

**Figure 1:**
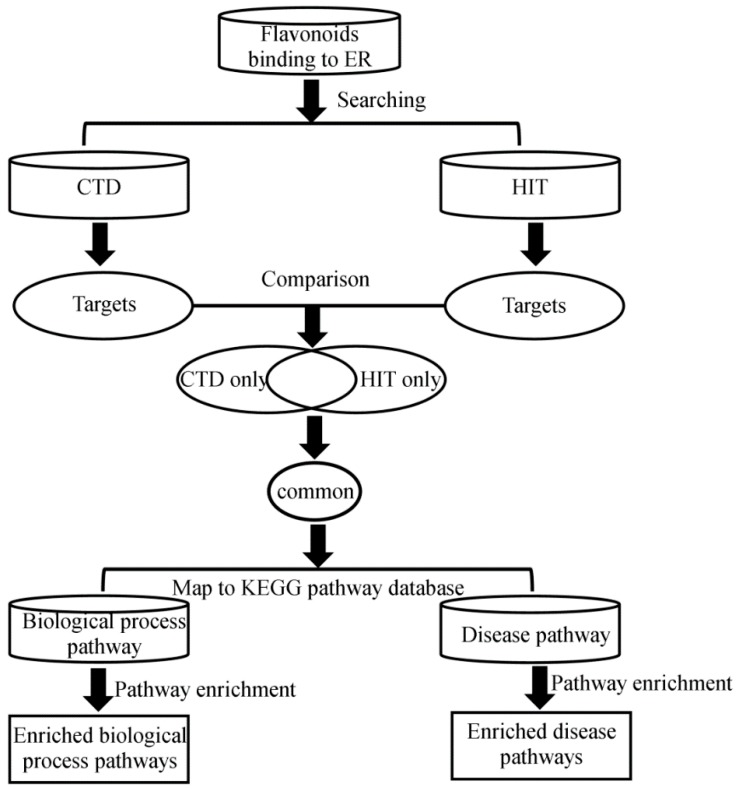
Overview of the study design.

Figure 1 depicts our study’s workflow. We retrieved from the EADB 22 flavonoids that exhibited estrogen receptor binding activity in our own validated relative binding affinity assays [39]. Then, direct binding targets of these flavonoids were taken from the Comparative Toxicogenomics Database (CTD) [40] and the Herbal Ingredients’ Targets (HIT) database [41]. In order to reduce the false positive rate, we used only the targets common to CTD and HIT. We then mapped the targets to biological process pathways and disease pathways in Kyoto Encyclopedia of Genes and Genomes (KEGG). Finally, the hypergeometric statistics model was used to identify the enriched pathways of the selected common targets.

### 2.2. Flavonoids with ER Binding Activity and Their Targets

**Figure 2:**
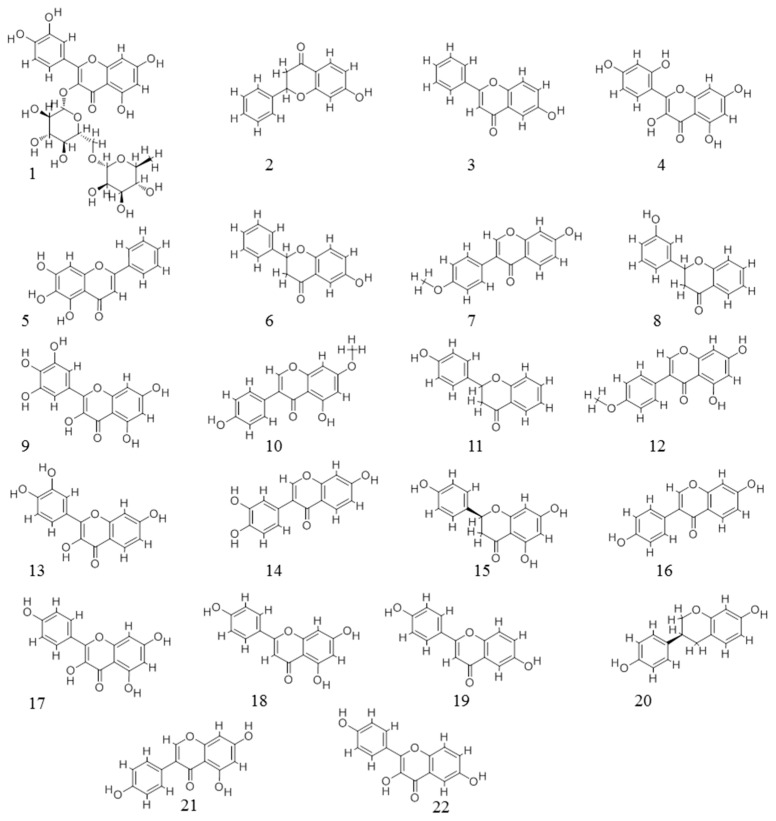
Structures of flavonoids.

We retrieved 22 flavonoid’s ER binding activity data from our earlier competitive binding assays [39]. Basic information of the 22 flavonoids—including Chemistry Abstract Service (CAS) number, structure, log (relative binding affinity)—is listed as Table 1. The structures of the 22 flavonoids are shown in Figure 2.

#### 2.2.1. Targets from CTD

CTD [40] is a comprehensive database of curated information on environment exposure effects on human health. It comprises three major categories of data: chemical-gene/protein, chemical-disease and gene-disease relationships. Data are the result of manual curation and text-mining of the literature. We carried out searches of the 22 flavonoids one by one in the CTD website [49]. Only the manually curated chemical-gene records with “binding interaction” were selected. From this, we retrieved 65 targets for 17 flavonoids in CTD that are listed in Table 2.

#### 2.2.2. Targets from HIT

HIT [41] target information came from manual curation of herbal ingredient chemicals from PubMed abstracts. It covers the relationships of herb-compound, compound-gene, and Traditional Chinese Medicine (TCM) formula-herb. With respect to compound-gene relationships, the targets were divided into indirect targets and direct targets based on the binding information. In this study, we retrieved the direct targets information through keyword search of the 22 flavonoids at the HIT website [50]. Finally, 74 direct targets were retrieved for 11 flavonoids contained in HIT, as listed in Table 3.

### 2.3. Pathway Data

The KEGG [42] pathway database contains a series of manually drawn pathway maps representing our current knowledge on molecular interactions. It is one of most frequently used reference knowledge databases for gene function annotation. The KEGG pathway database contains two major types of pathways: biological process pathways (including metabolism, genetic information processing, environmental information processing, cellular processes, and organismal systems) and human disease pathways. In this study, we downloaded the XML files of each pathway map. Then, python scripts were developed to parse the genes in the pathway maps from the original XML files. In the end, we collected 223 biological process pathways and 71 human disease pathways, covering 6088 genes and 2459 genes, respectively (updated by 22th October 2015), for use in pathway enrichment calculations.

### 2.4. Pathway Enrichment Analysis

Pathway enrichment analysis is the predominant approach used for deciphering the biological functions associated with a list of genes [43]. In this study, the targets of estrogenic flavonoids common to CTD and HIT were used as the input gene list to detect the potential biological functions that might be influenced by flavonoids that are ER binders. The targets were first mapped to the biological process pathways and the human disease pathways. Then pathway enrichment was calculated on the pathways containing common targets. The enrichment calculation used the hypergeometric statistical model to identify the enriched pathways in the manner described in our previously reported studies [44,45,46,47].

For each pathway i, we calculated a *p*-value using Equation (I) and an enrichment factor (EF) using Equation (II).

(I) $$p=1-\sum_{x=0}^{x=a}\frac{\left(\begin{array}{c} n\\ x \end{array}\right)*\left(\begin{array}{c} M-n\\ a-x \end{array}\right)}{\left(\begin{array}{c} M\\ a \end{array}\right)}$$

(II) EF=anAM

where *a* is the number of common targets associated with pathway i; *n* is the number of all genes in pathway i; *M* indicates the number of all genes associated with all pathways containing the common targets*;* and *A* is the number of all common targets covered by the pathways.

In the enrichment analysis, the criteria *a* ≥ 2 (the enriched pathways contain at least two common targets), EF ≥ 3 and *p*-value ≤ 0.05 were used to designate enriched pathways.

## 3. Results

### 3.1. Common Targets

Table 4 lists the common flavonoids target hits for CTD and HIT (for the 22 flavonoids and 14 target genes common to both databases). Cytochrome CYP450 family and nuclear hormone receptors are the two major gene families associated with targets. As expected, genes ESR1 and ESR2 were among the common targets, considering that all of the 22 flavonoids are ER binders. CYP450 genes involve the metabolic process of flavonoids. Other targets provide clues for discovering new putative functions of the flavonoids.

### 3.2. Biological Process Pathways of the Common Targets

After mapping the 14 common targets to the 223 biological process pathways of KEGG, we found 11 common targets were associated with 35 biological process pathways. Among the 35 biological process pathways, eight were designated as enriched based our stated criteria, *i.e.*, two or more common targets, *p*-value ≤ 0.05 and EF ≥ 3. The pathways designated as enriched are listed in Table 5. Expectedly, given that all 22 flavonoids are ER binders, the estrogen signaling pathway where ER is the mediating receptor was enriched. The other enriched pathways suggest that flavonoid ER binders potentially affect other biological processes in three categories: ER regulated processes; estrogen metabolism and synthesis; and cellular apoptosis.

#### 3.2.1. ER Regulated Processes

This category contains two pathways: prolactin signaling pathway and tryptophan metabolism pathway. Prolactin is a polypeptide hormone that binds to prolactin receptors. The expression of prolactin is modulated by estrogen [48]. The 22 flavonoids are ER binders and thus impact the prolactin signaling pathway like estrogens through modulating prolactin expression. Since ER is known to regulate tryptophan hydroxylase [51], it is rational that the subsequent biological process of ER binders incorporates tryptophan metabolism. In addition, tryptophan is a precursor to the neurotransmitters serotonin and melatonin [52]. Flavonoids with ER-binding activity may also influence the biological processes related to neurotransmitters serotonin and melatonin such as the circadian clock controlled by melatonin [53]. In this pathway, two common targets (CYP1A1, CYP1B2) are directly involved in the metabolism of melatonin.

#### 3.2.2. Estrogen Metabolism and Synthesis

This category consists of three pathways: ovarian steroidogenesis, steroid hormones biosynthesis, and metabolism of xenobiotics by cytochrome CYP450. Ovarian steroidogenesis comprises two processes: converting cholesterol to androgens and transforming androgens into estrogens. Since the 22 flavonoids are ER binders, they may influence the concentration balances between *endogenous estrogens and androgens* [54]. In this pathway, *CYP1A1*, *CYP1B1* and *CYP19A1* are involved in the biosynthesis of estrogens. Steroid hormone biosynthesis [55] is the process of generating three groups of steroids (C21, C19, C18) from cholesterol, in two major steps. In the first step, the C27 compound cholesterol is cleaved into progestogens (C21) by cholesterol side-chain cleavage enzymes. In the second step, progestogens are used for synthesis of glucocorticoids and mineralocorticoids (C21), androgens (C19), and estrogens (C18). The flavonoids with ER-binding activity may influence the biosynthesis of endogenous estrogens through *CYP1A1, CYP1B1 and CYP19A1*. Generally, estrogens are removed from the body through transformation into estrogenically inactive metabolites. The concentration of estrogens could be regulated by the common targets (CYP1A1 and CYP1B1) in this pathway. Specifically, the estrogenically inactive metabolites of 2-hydroxyestradiol and 4-hydroxyestradiol from estrogens are catalyzed by CYP1A1 and CYP1B1, respectively [56].

#### 3.2.3. Cellular Apoptosis

The last category of pathways involves cellular apoptosis. Estrogen’s role in regulating apoptosis has strong evidence [57,58]. In fact, using high-dose synthetic estrogens for breast cancer has been an effective therapy for four decades. Blocking the endogenous estrogen binding to ER with the antagonist Tamoxifen is another prevalent breast cancer treatment [59]. Tumor necrosis factor (TNF) could induce apoptosis [60] and activate the estrogen signaling pathway [61]. Flavonoids with ER-binding activity may influence cellular apoptosis through Casp3 and TNF-alpha.

### 3.3. Disease Pathways of the Common Targets

We also investigated the potential disease pathways influenced by the flavonoids’ common targets. After mapping the 14 common targets to the 71 human disease pathways, we found nine common targets that could be involved in the disease pathways. In total, 42 disease pathways contained the common targets of the 22 flavonoids. Table 6 lists 10 disease pathways designated as enriched, having at least two flavonoid targets, with a *p*-value ≤ 0.05 and EF ≥ 3. Interestingly, most of them were involved in five disease types: cancer, infectious diseases, neurodegenerative diseases, cardiovascular diseases, endocrine and metabolic diseases.

#### 3.3.1. Cancer

Several epidemiological studies [4,62,63] strongly support flavonoid rich diets as reducing cancer. There are many *in vitro* and animal experimental data showing associations between cancer risk and flavonoid chemicals such as quercetin [64] and (-)-Epigallocatechin-3-gallate [65]. However, the role of flavonoids in cancer risk remains uncertain as there are very few data in humans available [66]. In the pathway map of “Proteoglycans in cancer”, flavonoids may influence cancer through three different signaling paths. Flavonoids targeting ER could modulate the gene expression of cyclin D1, which could finally control cell growth and survival. In addition, flavonoids can directly control cell apoptosis through its target Casp3. Regulating cell growth through TNF-alpha and CAV1 is another way that flavonoids might affect cancer. In the chemical carcinogenesis process, flavonoids could influence the metabolism of aromatic hydrocarbons, azo-dyes and olefines through CYP1A1 and CYP1B1.

#### 3.3.2. Infectious Diseases

In response to bacterial or parasitic infections, apoptosis would be initiated by the host cells. Such innate immune response could remove the external pathogens at the early stage of infection. Flavonoids could modulate apoptosis by targeting Casp3 in the infection process of pertussis, legionellosis, amoebiasis, toxoplasmosis and hepatitis B. In addition, during the subsequent inflammation process followed by infection, flavonoids could regulate inflammation through TNF-alpha. Some flavonoids also have antimicrobial effects, such as antibacterial [67] and antifungal [68]. For example, oral administration of tryptanthrin and/or kaempferol significantly decreased the numbers of colonies of helicobacter pylori in helicobacter pylori-infected Mongolian gerbils [69].

#### 3.3.3. Neurodegenerative Diseases

Amyotrophic lateral sclerosis (ALS) is a fatal motor neuron disease, causing muscle weakness and atrophy throughout the body. Astrocytes and microglias are involved the neurodegenerative process of ALS [70,71]. Flavonoids could regulate the apoptosis of astrocyte through Casp3. In addition, the growth and death of microglial cell could be regulated by flavonoids targeting TNF-alpha. Some polyphenols may have protective effects against cognitive decline [72] and other neurodegenerative diseases. For example, curcumin and apigenin were proposed as promising therapeutics to slow down the progression of Alzheimer’s disease [73].

#### 3.3.4. Cardiovascular Diseases

Epidemiological evidence on the reduction of the CVD risk in high flavonoid diets is very consistent [74]. In a 7-year-follow-up prospective cohort study on 98,469 U.S. adults, five flavonoid chemicals (anthocyanins, flavan-3-ols, flavones, flavonols, and proanthocyanidins) were found to be individually associated with lower risk of fatal CVD [9]. Viral Myocarditis is a cardiac disease characterized by heart muscle inflammation, as a consequence of viral infection [75]. Flavonoids could modulate the death of infected myocytes via Casp3 and CAV1 in the early stage of viral infection.

#### 3.3.5. Endocrine and Metabolic Diseases

Non-alcoholic fatty liver disease (NAFLD) is a chronic liver disease associated with lipid deposition and inflammation in hepatocytes. Insulin resistance is an important factor for progressive steatohepatitis in NAFLD [76]. Flavonoids could influence the hepatocyte insulin resistance via TNF-alpha. In addition, the apoptosis of injured hepatocytes could be induced by flavonoids targeting Casp3. There is also a lot of evidence of the protective role of flavonoids against diabetes [77]. Oral administration of rutin (100 mg/kg) to diabetic rats for a period of 45 days decreased plasma glucose and increased insulin level by altering glycolytic and gluconeogenic enzymes [78]. Treatment with isoquercetin at dose 200 mg/kg was found to decrease blood glucose concentration in diabetic KK-Ay mice [79].

## 4. Discussion

Chemicals exert their biological functions through binding or interacting with the targets. The overall target characteristics of flavonoids that bind ER may provide clues for more fully delineating their risk *versus* benefit profiles. Identifying the targets beyond ER itself is the starting place. Molecular docking [80,81,82,83], machine learning [84,85], text mining and network inference [86,87] are among the many sound computational methods for predicting alternate targets of chemicals. However, a high false positive rate is a side effect of most approaches, and a main reason we chose to use only manually curated targets from CTD and HIT. While the more meager data increasing the likelihood of missing flavonoid pathway associations, we deemed developing a more trustworthy association profile more important. Among the 22 estrogenic flavonoids we included, only 11 and 17 of them are covered by HIT and CTD, respectively, with 14 common flavonoid targets. The commonality restriction we imposed enhances true positive rate and the validity of the final pathway association profile. Even with the more rigorous screening of the data, abundant and biologically rational conclusions were made with no contradictions to existing science.

Pathways are manually curated series of molecular interactions that encode diverse research results that converge and elucidate a complex biological process connecting molecular entities and events. As long as more and better data are forthcoming, pathways will increasingly encompass the vital interconnections that are the basis of life. Pathway enrichment analyses provide a means for disentangling more accurately the true biology in a system so complex that false random solutions abound. In our present work, we have applied reasoning to filter data to increase actual cause and effect and reduce random correlations, in this case to help reveal the multiple biological meanings and actions associated with flavonoids, based on knowledge about their molecular targets. According to our pathway enrichment results, CYP1A1, CYP1B1, CYP19A1, Casp3, TNF-alpha are genes associated with multiple pathways having substantial cross talk. For example, CYP1A1, CPY1A2 could participate in estrogen metabolism and biosynthesis as well as tryptophan metabolism. Casp3’s and TNF-alpha’s apoptosis suggest their affiliation with the pathological development of across several diseases. Flavonoids could regulate the cell death of astrocytes and microglias in brain, myocytes in heart, as well as cancer cells or abnormal or injured cells. Given that apoptosis is ubiquitous in biological process across cell types, risks incurred with flavonoid doses higher than what humans have evolved with should be concerning and more carefully studied. Indeed, several identified pathways based on targets in CTD and HIT had already been validated by previous studies of flavonoids compounds. For example, apigenin can induce the apoptosis process of NCI-H460 cells by up-regulating expression of Bax and Casp3 and down-regulating the expression of Bcl-2 [88]. Quercetin was shown to significantly inhibit hepatitis B virus replication and hepatitis B surface antigen secretion in human hepatoma cell lines: HepG2.2.15 cell and HuH-7 cell [89]. (-)-Epigallocatechin-3-gallate (EGCG) displayed the neuroprotective effects on the transgenic mouse model of amyotrophic lateral sclerosis [90].

In addition, co-incubation with lamivudine (3TC), entecavir (ETV), or adefovir (Ade) further enhanced the quercetin-induced inhibition of Hepatitis B virus (HBV) replication. This inhibition was partially associated with decreased heat shock proteins and HBV transcription levels. The results indicate that quercetin inhibited HBV antigen secretion and genome replication in human hepatoma cell lines, which suggests that quercetin may be a potentially effective anti-HBV agent.

## 5. Conclusions

Here, we used pathway enrichment analysis on carefully vetted data to derive a profile of potential risk associated with 22 flavonoids that bind highly conserved estrogen receptor nuclear proteins. To improve the likelihood of enriched pathways with true positives, we restricted data to 14 targets of flavonoids identified by both CTD and HIT manually curated databases. Enriched pathways of targets were found to be mainly involved in three paramount biological process types, ER regulated processes, estrogens’ metabolism and synthesis, and as well as apoptosis. Among the findings is a cause for concern that flavonoids via apoptosis pathways could be especially important for cancer, infection, amyotrophic lateral sclerosis, viral myocarditis and non-alcoholic fatty liver disease. Further attention should be paid to potential apoptosis effects of flavonoids that bind ERs. The approach we present should provide ever improving clues to potential risk posed by the important flavonoids that have long been a component of human food, and that is now prominent in food supplements at doses far above historical norms.

## Figures and Tables

**Table 1:** Basic information of flavonoids with estrogen receptor (ER) binding activity.

ID	Name	CAS	ER Activity	Number of Targets	
				HIT	CTD
1	Rutin	153-18-4	−4.09	3	5
2	7-Hydroxyflavanone	6515-36-2	−3.73	0	2
3	6-Hydroxyflavone	6665-83-4	−3.41	0	1
4	Morin	480-16-0	−3.09	4	1
5	Baicalein	491-67-8	−3.05	4	4
6	6-Hydroxyflavanone	4250-77-5	−3.05	0	0
7	Formononetin	485-72-3	−2.98	2	3
8	3-hydroxyflavanone	92496-65-6	−2.78	0	2
9	Myricetin	529-44-2	−2.75	9	2
10	Prunetin	552-59-0	−2.74	0	0
11	4-hydroxyflavanone	6515-37-3	−2.65	0	3
12	Biochanin A	491-80-5	−2.37	0	12
13	Fisetin	528-48-3	−2.35	8	2
14	3,4,7-Trihydroxyisoflavone isoflavone	485-63-2	−2.35	0	0
15	Naringenin	480-41-1	−2.13	4	12
16	Daidzein	486-66-8	−1.65	12	12
17	Kaempferol	520-18-3	−1.61	12	20
18	Apigenin	520-36-5	−1.55	15	16
19	4,6-Dihydroxyflavone	63046-09-3	−0.82	0	0
20	Equol	531-95-3	−0.82	0	7
21	Genistein	446-72-0	−0.36	16	37
22	3,6,4-Trihydroxyflavone	253195-19-6	−0.35	0	0

**Table 2:** Targets of flavonoids selected from the EADB retrieved from the CTD.

Gene (Entrz ID)	Chemical IDs	Gene (Entrz ID)	Chemical IDs
ABCC2 (1244)	21	FOXO3 (2309)	21
AFP (174)	2, 5, 8, 9, 11, 13, 15, 17, 18, 20	GPER1 (2852)	21
AHR (196)	5, 12, 15, 16, 17, 18, 21	GREB1 (9687)	17, 21
AIP (9049)	17, 18	HIF1A (3091)	21
AKR1C1 (1645)	15	HIST3H3 (8290)	21
AKT1 (207)	21	JUN (3725)	17, 18
ALB (213)	1, 7	JUND (3727)	21
ALPL (249)	15, 16, 21	MAP2K1 (5604)	9
APP (351)	4, 5, 17	NCOA1 (8648)	12, 21
AR (367)	3, 11, 13, 20, 21	NCOA2 (10499)	16, 21
ARNT (405)	15, 17, 18, 21	NCOA3 (8202)	17
BAX (581)	17	NFKBIA (4792)	18
BBC3 (27113)	17	NFKBIB (4793)	18
BCL2L1 (598)	17	NOS3 (4846)	20
CASP3 (836)	1	NR1H3 (10062)	21
CASP8 (841)	1	NR1I2 (8856)	16, 21
CAV1 (857)	20	NR3C1 (2908)	21
CBR1 (873)	1, 21	NRIP1 (8204)	21
CCND1 (595)	1	PGR (5241)	12, 17, 18
CDK2 (1017)	16	PNRC1 (10957)	12, 16, 21
CDKN1A (1026)	16	PPARG (5468)	17, 18
CYLD (1540)	21	PTEN (5728)	21
CYP19A1 (1588)	12, 15	RORC (6097)	12
CYP1A1 (1543)	12, 15, 17, 18	RXRA (6256)	21
CYP1B1 (1545)	5, 15, 17, 18, 21	SHBG (6462)	2, 8, 11, 15, 18, 20, 21
EDN1 (1906)	21	SI (6476)	15
ESR1 (2099)	7, 12, 15, 16, 17, 18, 20, 21	SIRT1 (23411)	21
ESR2 (2100)	7, 12, 15, 16, 17, 18, 20, 21	SP1 (6667)	21
ESRRA (2101)	12, 16, 21	SULT2A1 (6822)	21
ESRRB (2103)	12, 16, 21	TNF (7124)	17, 18
ESRRG (2104)	12, 16, 21	TP53 (7157)	17, 21
FOS (2353)	17, 18	TSC22D3 (1831)	21
		VDR (7421)	21

**Table 3:** Targets of flavonoids selected from the EADB retrieved from the HIT.

Gene (Entrz ID)	Chemical IDs	Gene (Entrz ID)	Chemical IDs
ADH1B (125)	16	GBA (2629)	21
ADH1C (126)	16	GSK3B (2932)	18
ADH4 (127)	16	HDAC1 (3065)	18
AHR (196)	17	HSD17B7 (51478)	18
AKTIP (64400)	9, 21	IL4 (3565)	13, 18
ALB (213)	21	JAK1 (3716)	9
ALG5 (29880)	18	LOX (4015)	5
ALOX12 (239)	5	MAP2K4 (6416)	21
ALOX5 (240)	4	MGAM (8972)	16
APEX1 (328)	21	MMP1 (4312)	17
AR (367)	13, 21	MMP2 (4313)	9
ATP1A1 (476)	9	MPO (4353)	1
AURKB (9212)	13	MTOR (2475)	13
BCHE (590)	15	NOS2 (4843)	5, 17
BDNF (627)	16	NR1I2 (8856)	16
BTK (695)	21	OLR1 (4973)	18
CASP3 (836)	9, 16	PARP1 (142)	9, 13
CAV1 (857)	16	PIP4K2A (5305)	4
CD38 (952)	18	PKD2 (5311)	15
CDK4 (1019)	13	PPARA (5465)	21
CDK9 (1025)	18	PPARG (5468)	17
CFTR (1080)	21	PPP3CA (5530)	17
COMT (1312)	9	PROCR (10544)	1
CXCR4 (7852)	18	PTK2B (2185)	16
CYP19A1 (1588)	15, 18	RAD51 (5888)	5
CYP1A1 (1543)	17	RELA (5970)	17
CYP1A2 (1544)	17	RPS9 (6203)	18
CYP1B1 (1545)	17	SCD (6319)	18
DNMT1 (1786)	21	SIRT1 (23411)	13
DPP4 (1803)	18	SLC2A1 (6513)	21
EGFR (1956)	21	SLC52A1 (55065)	16
EPHB2 (2048)	16	STAT3 (6774)	9
ESR1 (2099)	7, 9, 15, 16, 17, 21	SYK (6850)	21
ESR2 (2100)	7, 17, 21	TNF (7124)	1, 17
FABP4 (2167)	18	TOP1 (7150)	13
FAS (355)	4	VEGFA (7422)	18
FASN (2194)	21	XDH (7498)	4

**Table 4:** Common targets to CTD and HIT.

Target	Entrz Gene ID	Gene Family
CYP1A1	1543	Cytochrome P450 family 1
CYP1B1	1545	Cytochrome P450 family 1
CYP19A1	1588	Cytochrome P450 family 19
AR	367	Nuclear hormone receptors
ESR1	2099	Nuclear hormone receptors
ESR2	2100	Nuclear hormone receptors
NR1I2	8856	Nuclear hormone receptors
PPARG	5468	Nuclear hormone receptors
TNF	7124	Tumor necrosis factor superfamily
ALB	213	Unglycosylated serum protein
AHR	196	Basic helix-loop-helix proteins
CASP3	836	Caspases
CAV1	857	Membrane proteins
SIRT1	23411	Sirtuins

**Table 5:** Enriched biological process pathways.

Pathway	Category	EF	*p* Value
Tryptophan metabolism	Amino acid metabolism	8.3203	0.0014
Apoptosis	Cell growth and death	4.0634	0.0110
Ovarian steroidogenesis	Endocrine system	2.7301	0.0321
Prolactin signaling pathway	Endocrine system	10.2781	0.0001
Estrogen signaling pathway	Endocrine system	4.8535	0.0067
Steroid hormone biosynthesis	Lipid metabolism	3.5659	0.0302
TNF signaling pathway	Signal transduction	2.9615	0.0426
Metabolism of xenobiotics by P450	Xenobiotics biodegradation & metabolism	2.5508	0.0383

**Table 6:** Enriched disease pathways.

Pathway	Category	EF	*p* Value
Proteoglycans in cancer	Cancers	4.0672	0.0013
Chemical carcinogenesis	Cancers	5.5127	0.0045
Pertussis	Infectious diseases: Bacterial	6.1832	0.0032
Legionellosis	Infectious diseases: Bacterial	8.3192	0.0014
Amoebiasis	Infectious diseases: Parasitic	4.3166	0.0089
Toxoplasmosis	Infectious diseases: Parasitic	3.4403	0.0166
Hepatitis B	Infectious diseases: Viral	3.0916	0.0222
Amyotrophic lateral sclerosis	Neurodegenerative diseases	8.6331	0.0012
Viral myocarditis	Cardiovascular diseases	6.3549	0.0030
Non-alcoholic fatty liver disease	Endocrine and metabolic diseases	3.0302	0.0234

## Abbreviations

The following abbreviations are used in this manuscript:

ABC	ATP-Binding Cassette
ALS	Amyotrophic Lateral Sclerosis
CTD	Comparative Toxicogenomics Database
EADB	Estrogenic Activity Database
EDSP	Endocrine Disruptor Screening Program
EMA	European Medicines Agency
EPA	Environmental Protection Agency
ER	Estrogen Receptor
FDA	Food and Drug Administration
HIT	Herbal Ingredients’ Target
NAFLD	Non-alcoholic Fatty Liver Disease
KEGG	Kyoto Encyclopedia of Genes and Genomes
EGCG	(-)-Epigallocatechin-3-Gallate
